# Japanese value set for the EORTC QLU-C10D: A multi-attribute utility instrument based on the EORTC QLQ-C30 cancer-specific quality-of-life questionnaire

**DOI:** 10.1007/s11136-024-03655-7

**Published:** 2024-05-09

**Authors:** T. Shiroiwa, M. T. King, R. Norman, F. Müller, R. Campbell, G. Kemmler, T. Murata, K. Shimozuma, T. Fukuda

**Affiliations:** 1 Center for Outcomes Research and Economic Evaluation for Health (C2H), National Institute of Public Health, Wako, Saitama Japan; 2 Faculty of Science, School of Psychology, University of Sydney, Sydney, NSW Australia; 3 European Organisation for Research and Treatment of Cancer Quality of Life Group, Brussels, Belgium; 4 School of Population Health, Curtin University, Perth, WA Australia; 5 Medical Psychology, Amsterdam UMC Location University of Amsterdam, Meibergdreef 9, Amsterdam, Netherlands; 6 Amsterdam Public Health, Global Health, Amsterdam, Netherlands; 7 Department of Psychiatry, Psychotherapy and Psychosomatics I, Medical University of Innsbruck, Innsbruck, Austria; 8 Crecon Medical Assessment Co., Ltd, Tokyo, Japan; 9 College of Life Sciences, Ritsumeikan University, Kusatsu, Japan

**Keywords:** Multi-attribute utility, Preference-based measure, Cancer, Discrete choice experiment, European Organisation for Research and Treatment of Cancer (EORTC) Quality of Life Questionnaire-Core 30 items, QLQ-C30, Quality of Life Utility Core 10 dimensions, QLU-C10D

## Abstract

**Purpose:**

This study aimed to develop a Japanese value set for the EORTC QLU-C10D, a multi-attribute utility measure derived from the cancer-specific health-related quality-of-life (HRQL) questionnaire, the EORTC QLQ-C30. The QLU-C10D contains ten HRQL dimensions: physical, role, social and emotional functioning, pain, fatigue, sleep, appetite, nausea, and bowel problems.

**Methods:**

Quota sampling of a Japanese online panel was used to achieve representativeness of the Japanese general population by sex and age (≥ 18 years). The valuation method was an online discrete choice experiment. Each participant considered 16 choice pairs, randomly assigned from 960 choice pairs. Each pair included two QLU-C10D health states and life expectancy. Data were analyzed using conditional logistic regression, parameterized to fit the quality-adjusted life-year framework. Preference weights were calculated as the ratio of each dimension-level coefficient to the coefficient for life expectancy.

**Results:**

A total of 2809 eligible panel members consented, 2662/2809 (95%) completed at least one choice pair, and 2435/2662 (91%) completed all choice pairs. Within dimensions, preference weights were generally monotonic. Physical functioning, role functioning, and pain were associated with the largest utility weights. Intermediate utility weights were associated with social functioning and nausea; the remaining symptoms and emotional functioning were associated with smaller utility decrements. The value of the worst health state was − 0.221, lower than that seen in most other existing QLU-C10D country-specific value sets.

**Conclusions:**

The Japan-specific QLU-C10D value set is suitable for evaluating the cost and utility of oncology treatments for Japanese health technology assessment and decision-making.

**Supplementary Information:**

The online version contains supplementary material available at 10.1007/s11136-024-03655-7.

## Plain English summary

1. Why is this study needed?

The EORTC QLU-C10D is a preference-based multi-attribute utility instrument (MAUI) derived from the EORTC Quality of Life Questionnaire-Core 30 (QLQ-C30), a health-related quality-of-life (HRQL) questionnaire widely used in cancer clinical trials internationally. The QLU-C10D enables quantification of utility from responses to the QLQ-C30, and hence enables HRQL data to be used in health policy decisions about cancer. Such decisions are typically made within a country, relating to the health budget of that country, or of regional and local health authorities. Therefore, country-specific ‘value sets’ based on the values and preferences of the general population of specific countries are needed.

2. What is the key problem/issue/question this manuscript addresses?

No value sets for the EORTC QLU-C10D existed for Japan prior to this study.

3. What is the main point of your study?

The valuation survey used to develop the Japanese QLU-C10D value set followed the standard protocol developed by the Multi-Attribute Utility in Cancer (MAUCa) Consortium for evaluating the EORTC QLU-C10D in general population samples. The valuation method used was an online discrete choice experiment. The resultant value set enables HRQL data from the EORTC QLQ-C30 to be used in Japanese health policy decisions and health technology assessment.

4. What are your main results and what do they mean?

A Japanese value set for the EORTC QLU-C10D was created. Physical functioning, role functioning, and pain were associated with the largest utility weights. The value of the worst health state was -0.221, lower than that seen in most other existing QLU-C10D country-specific value sets.

## Introduction

When economic evaluation of healthcare technologies is performed, quality-adjusted life years (QALYs) are standardly used for outcome measurement. QALYs can be calculated by weighting life years by the utility of the health state[1]. In Japan, since 2019, economic evaluation submissions are required for selected drug and medical device pricing before the Ministry of Health, Labour and Welfare (MHLW) can approve higher prices than for existing drugs[2]. As of December 2022, evaluations of 39 drugs and devices were completed or are in progress. The guideline for submission to the authority [3] indicates that “QALY should be used in principle” and “If Japanese quality-of-life (QOL) scores (utilities) are newly collected for a cost-effectiveness analysis, EQ-5D-5L is recommended as the first choice.” However, it does not preclude the use of alternative utility instruments.

For the purposes of economic evaluation, utility is anchored as 0 = dead and 1 = full health, which is necessary for construction of QALYs. To obtain scores on this utility scale, we typically use a preference-based measure (PBM). Many generic PBMs have been developed to measure utility, for example, the EuroQol 5 Dimensions (EQ-5D) [4, 5], Health Utilities Index (HUI)[6, 7], and Short Form 6 Dimensions (SF-6D) [8]. On the other hand, in clinical studies, disease-specific profile-type instruments are often used to measure patients’ HRQL. However, profile-type measures cannot be used for economic evaluation because they are not preference-based and therefore do not measure utility.

In collaboration with the European Organisation for Research and Treatment of Cancer (EORTC) Quality of Life Group, the Multi-Attribute Utility in Cancer (MAUCa) Consortium has developed the EORTC QLU-C10D, a multi-attribute utility instrument (MAUI) derived from the EORTC Quality of Life Questionnaire-Core 30 (QLQ-C30) [9, 10]. The QLQ-C30 is the most widely used cancer-specific HRQL questionnaire [11]. But because the QLQ-C30 is a profile-type measure, it cannot be used to quality-adjust survival to calculate QALYs. The QLU-C10D was developed to enable quantification of utility from responses to the QLQ-C30. While mapping algorithms are available to derive utilities from QLQ-C30 responses through generic MAUIs [12], the QLU-C10D is potentially theoretically and empirically stronger because it comprises a descriptive system and a valuation method that complies with the Checklist for Reporting Valuation Studies [13], and aims to retain the cancer-specific sensitivity which is part of the QLQ-C30. Five of the 10 QLU-C10D dimensions capture symptoms and impacts of cancer and its treatments that are not explicitly included in generic instruments: nausea, fatigue, loss of appetite, and problems with sleep and bowel function. The other five dimensions are pain and four aspects of functioning (physical, role, social, and emotional). The QLU-C10D is not a stand-alone questionnaire; it is a MAUI that comprises a health state descriptive system plus country-specific preference weighting algorithms. Online Resource 1 shows the QLU-C10D descriptive system and explains how the 10 dimensions can be derived from 13 of the 30 items in the QLQ-C30.

The MAUCa Consortium has developed a standard protocol for evaluating the EORTC QLU-C10D in general population samples, as described. Using this method, value sets have been estimated for 11 countries so far, with more in progress [14–22]. This study aimed to apply this valuation method in a Japanese general population sample to produce Japan-specific utility weights and value set for the QLU-C10D, and to compare the Japanese value set to those from other countries.

## Methods

A cross-sectional population-based survey was designed to collect QLU-C10D valuation data from a representative sample of the Japanese general population; the study protocol was approved by the Japanese National Institute of Public Health ethics committee (approval number NIPH-IBRA #12272). The methods were consistent with previous QLU-C10D valuation studies [18, 19, 21, 23–28].

The survey was implemented by SurveyEngine, a company specialized in online choice experiments. SurveyEngine managed sample recruitment (via a Japanese online panel), survey administration, and data collection. SurveyEngine and its panel provider complied with the International Code on Market, Opinion and Social Research and Data Analytics [29]. The survey opened on 5th February 2021 and closed on 15th March 2021. Online panel members were eligible if they were aged ≥ 18 years and able to read and understand Japanese. Online panelists received an e-mail invitation to participate, including a hyperlink to the study and survey. Panel members who attempted to enter the survey via mobile phones were screened out as the discrete choice experiment (DCE) was too complex for a small screen. Consent was sought from the remainder, who were screened for quota sampling to ensure the age and sex distributions of the sample matched those of the Japanese general population (Table 1). Participants who consented and were within quota proceeded to further survey questions.

**Table 1:** Self-reported sociodemographic characteristics and health of the Japanese valuation survey sample (n =  2435 participants who completed 16 Discrete Choice Experiment choice sets) compared with the Japanese general population

Characteristics	Category		Sample, n	Sample, % or mean, $\overline{{\varvec{x}} }$	Japanese population, %	Test statistic^a^	*p* value
Sex^b^	Male		1158	47.6%	48.7%	*X*^2^ = 1.27	0.259
	Female		1277	52.4%	51.3%		
Age^b^	18–29 years		322	13.2%	14.0%	*X*^2^ = 5.44	0.364
	30–39 years		314	12.9%	13.3%		
	40–49 years		405	16.6%	17.2%		
	50–59 years		360	14.8%	15.1%		
	60–69 years		372	15.3%	15.1%		
	70 years or older		662	27.2%	25.3%		
Region^b^	Hokkaido		122	5.1%	4.2%	*X*^2^ = 105.64	< .001
	Tohoku		128	5.4%	7.0%		
	Kanto		975	40.8%	34.2%		
	Chubu		379	15.9%	16.8%		
	Kansai		461	19.3%	17.7%		
	Chugoku		109	4.6%	5.8%		
	Shikoku		55	2.3%	3.0%		
	Kyushu		160	6.7%	11.4%		
	Missing		46	–			
Work^e^	Engaged mainly in work		1204	49.4%	49.8%	*X*^2^ = 276.61	< .001
	Engaged in work while attending school/housekeeping		265	10.9%	9.2%		
	Not at work		68	2.8%	1.6%		
	Unemployed person		45	1.8%	1.5%		
	Attending school		43	1.8%	5.4%		
	Housekeeping		426	17.5%	12.0%		
	Other		384	15.8%	20.5%		
	Missing		–				
Paid employment^c^	Full time worker		841	52.5%	54.4%	*X*^2^ = 6.16	.104
	Part-time worker		386	24.1%	23.6%		
	Temporary worker		167	10.4%	8.8%		
	Director/Self-employer		208	13.0%	13.3%		
Household income^d^	< JPY 1million		169	7.1%	6.4%	*X*^2^ = 113.38	< .001
	JPY 1 million < = < JPY 2 million		185	7.8%	12.6%		
	JPY 2 million < = < JPY 3 million		320	13.4%	13.6%		
	JPY 3 million < = < JPY 4 million		360	15.1%	12.8%		
	JPY 4 million < = < JPY 5 million		354	14.8%	10.5%		
	JPY 5 million < = < JPY 7 million		401	16.8%	16.7%		
	JPY 7 million < = < JPY 10 million		318	13.3%	15.2%		
	JPY 10 million < = < JPY 15 million		181	7.6%	8.8%		
	JPY 15 million < = < JPY 20 million		65	2.7%	2.1%		
	JPY 20 million <		33	1.4%	1.2%		
	Missing		49	–	–		
Education^e^	Elementary or Junior high school		50	2.1%	14.8%	*X*^2^ = 953.59	< .001
	High school		689	28.8%	39.7%		
	College		469	19.6%	20.5%		
	University or graduate		1181	49.4%	24.3%		
	Missing		46	–	–		
Relationship status^f^	Unmarried		705	30.4%	31.6%	*X*^2^ = 66.68	< .001
	Married		1351	58.2%	61.3%		
	Bereaved		113	4.9%	3.2%		
	Divorced		152	6.5%	3.9%		
	Missing		114	–	–		
Kessler 6 psychological distress scale^g^	0–4 (best)		1470	63.4%	71.0%	*X*^2^ = 160.97	< .001
	5–9		462	19.9%	18.7%		
	10–14		237	10.2%	7.6%		
	15–24 (worst)		149	6.4%	2.7%		
	Missing		117	–			
EQ-5D-5L visual analogue scale (VAS)^h^	Age	Gender					
	20–29	Male	156	$\overline{x }=$ 75.5	$\mu$= 82.6	*t* = − 4.32	< .001
		Female	152	$\overline{x }=$ 66.4	$\mu$= 81.2	*t* = − 7.68	< .001
	30–39	Male	158	$\overline{x }=$ 72.7	$\mu$= 79.3	*t* = − 4.58	< .001
		Female	152	$\overline{x }=$ 71.0	$\mu$= 79.4	*t* = − 5.41	< .001
	40–49	Male	205	$\overline{x }=$ 71.4	$\mu$= 78.8	*t* = − 5.51	< .001
		Female	194	$\overline{x }=$ 73.4	$\mu$= 80.1	*t* = − 4.84	< .001
	50–59	Male	179	$\overline{x }=$ 75.2	$\mu$= 77	*t* =− 1.38	.171
		Female	171	$\overline{x }=$ 76.4	$\mu$= 79.2	*t* = − 1.89	.061
	60–69	Male	173	$\overline{x }=$ 77.5	$\mu$= 77.3	*t* = 0.15	.881
		Female	173	$\overline{x }=$ 78.2	$\mu$= 80.5	*t* = − 1.86	.065
	70–79	Male	224	$\overline{x }=$ 80.0	$\mu$= 74.9	*t* = 5.09	< .001
		Female	300	$\overline{x }=$ 78.3	$\mu$= 76.9	*t* = 1.37	.171
	80–89	Male	15	$\overline{x }=$ 76.7	$\mu$= 70.3	*t* = 2.61	< .05
		Female	41	$\overline{x }=$ 71.5	$\mu$= 68.1	*t* = 1.06	.297
	Missing		142	–			
General health question^i^	Excellent [5]		125	8.6%			
	Very good [4]		522	32.2%			
	Good [3]		796	32.7%			
	Fair [2]		783	21.4%			
	Poor [1]		209	5.1%			
Total sample		2435					

^a^For categorical variables, the Chi-Squared Goodness-of-Fit test was used to compare observed category frequencies with those expected based on population proportions. For continuous variables, t-test was used to compare sample means to population means

^b^2019 Population statistics data from https://www.e-stat.go.jp/stat-search/files?page=1&layout=datalist&toukei=00200524&tstat=000000090001&cycle=7&year=20190&month=0&tclass1=000001011679&result_back=1&tclass2val=0

^c^Survey data (provided by *n* = 1602 survey participants who endorsed one of these four categories) were compared to 2019 Population statistics data after adjusting the general population percentages to be proportional to the four categories for which we had data. The remainder were either not in paid employment or possibly missed this question. 2019 Labour force survey data from https://www.e-stat.go.jp/stat-search/files?page=1&layout=datalist&toukei=00200531&tstat=000000110001&cycle=7&year=20190&month=0&tclass1=000001040276&tclass2=000001040283&tclass3=000001040284&result_back=1&tclass4val=0

^d^2018 Japanese “Comprehensive Survey of Living Conditions” data from https://www.e-stat.go.jp/stat-search/files?page=1&layout=datalist&toukei=00450061&tstat=000001129675&cycle=7&tclass1=000001130605&tclass2val=0

^e^2017 Japanese employment status survey data from https://www.e-stat.go.jp/stat-search/files?page=1&layout=datalist&toukei=00200532&tstat=000001107875&cycle=0&tclass1=000001107876&tclass2=000001107877&tclass3val=0

^f^2015 Japanese national census data from https://www.e-stat.go.jp/stat-search/files?page=1&layout=datalist&toukei=00200521&tstat=000001080615&cycle=0&tclass1=000001089055&tclass2=000001089056&tclass3val=0

^g^2019 Japanese “Comprehensive Survey of Living Conditions” data from https://www.e-stat.go.jp/stat-search/files?page=1&toukei=00450061&tstat=000001141126

^h^Japanese Population Norms of EQ-5D-5L (Shiroiwa, Noto and Fukida [30])

^i^No normative data available

A target sample size of ≥ 2000 respondents was determined to provide acceptable precision for model parameter estimates, based on the MAUCa consortium’s extensive experience with DCE valuation surveys and the number of health state comparisons in the QLU-C10D DCE [31–34]. This sample size was larger than most similar studies [35], and meets the various rules of thumb outlined by de Bekker-Grob et al. [36].

### DCE valuation task

The feasibility of the implemented valuation task was previously established [10]. The valuation task involved choosing between pairs of hypothetical health states from the QLU-C10D; each pair formed a choice set. Online Resource 2 provides an example choice set from the Japanese survey. Each respondent was asked to consider 16 choice sets and indicate which health state they would prefer to live in until death. Each health state was described in terms of the ten dimensions of the QLU-C10D and a specified duration of survival (life years), which could take the values 1, 2, 5, or 10 years. Survival duration allowed the trade-off between QoL and life expectancy to be inferred, and enabled anchoring of utility scores at dead (zero life years) [31, 35]..

The QLU-C10D health state classification system has over a million possible health states (4^10^ = 1,048,576). To determine which of these to include in the DCE, we constructed a designed experiment of 960 choice sets that maximized statistical efficiency of the utility model parameter estimation. The DCE contained 12 attributes: 11 attributes for the 10 QLU-C10D dimensions because two attributes were used to represent physical functioning (long and short walk); survival duration was included as the twelfth attribute to enable estimation on a health utilities scale. Twelve attributes is a relatively large number for respondents to consider simultaneously, so we simplified the cognitive task in three ways [10]: (1) we constrained the number of QLU-C10D dimensions that differed between health states in any given choice set to four; (2) we highlighted in yellow the four dimensions that differed within a choice set; (3) for the physical functioning dimension, the descriptors for levels 2 and 3 are conceptually complex, so to aid respondent comprehension, the two items (‘long walk’ and ‘short walk’) were presented separately in the survey but scored as one 4-level dimension in the DCE design. We successfully used this approach in all previous QLU-C10D valuation surveys [18, 19, 21, 23–28], confirming feasibility across 8 languages and 11 countries.

The DCE used the same designed experimental as in previous QLU-C10D valuation studies; how it was constructed has been explained previously [18, 19, 21, 23–28]. The final DCE experimental design consisted of 960 choice sets, with an estimated D-efficiency of 90.4% relative to the best design with that level of overlap. There were three levels of randomization in the DCE component of the survey: (1) each respondent was randomized to answer 16 of 960 choice sets in the DCE design; (2) which option was presented as Situation A or Situation B was randomized within each choice set to mitigate any ordering bias; (3) the order of QLU-C10D dimensions was randomized for each person to prevent any order effect, with duration always presented as the last attribute.

### Other survey content

**Fig. 1:**
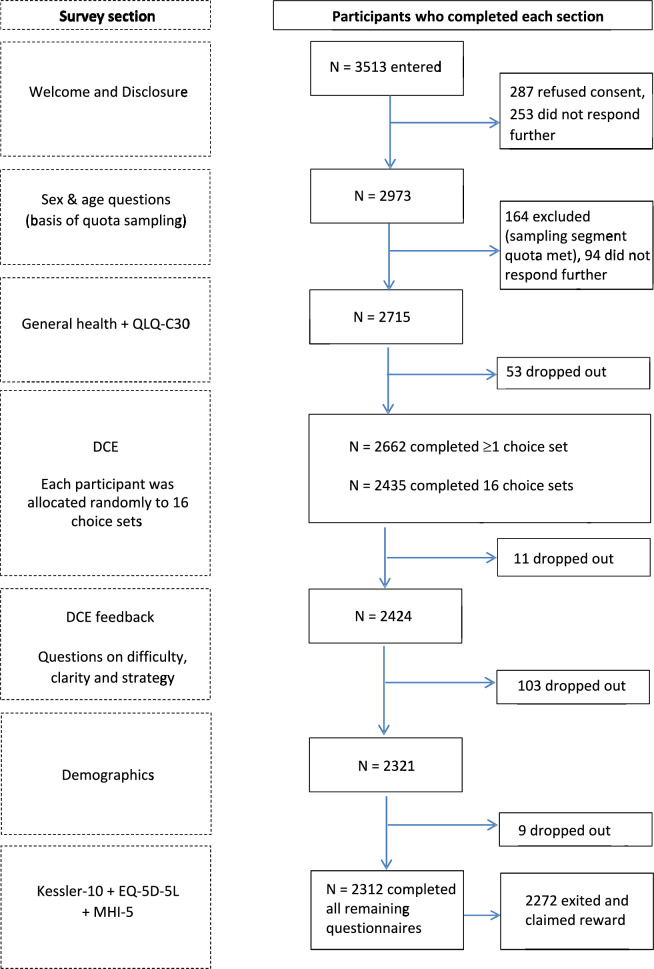
Respondent flow and sample sizes for each component of the valuation survey

The survey included several other components in the order shown in Fig. 1. These included sociodemographic characteristics and four validated self-reported health measures: the general health question from the 36-Item Short Form Health Survey (SF-36) [39], the EORTC QLQ-C30 [40], the Kessler 6 Psychological Distress Scale [41], and a preference-based generic health status measure, the 5-level version of the EQ-5D (EQ-5D-5L) [42, 43] After completing the DCE component, participants were asked four fixed-format questions about the difficulty and clarity of the valuation task and the strategy used to choose between health states (Online Resource 3).

### Statistical analyses

Descriptive statistics summarized sample demographics, self-reported general health, and participant feedback on the DCE valuation task. Sample representativeness was assessed against population reference data for demographics and self-reported general health using chi-square tests and t-tests.

Analysis of the DCE data followed the MAUCa consortium’s standard approach, as described previously for other QLU-C10D country-specific value sets [18, 19, 21, 23–26, 28]. This yields utility estimates consistent with standard QALY model restrictions by using a functional form we and others have used previously [10, 33, 34, 43, 44]. The QALY model requires all health states have zero utility at dead [45]. This requirement is satisfied by Eq. 1 and 2 because they include the interaction between the QLU-C10D levels and a *TIME* variable representing survival duration (life years). The designed experiment allowed for all these interactions. In Eqs. 1 and 2, as *TIME* tends to zero, the systematic component of the utility function tends to zero. Another requirement of the QALY model is constant proportional time trade off, therefore the relationship between utility and *TIME* (life years) was considered to be linear.

A useful feature of this functional form is that the impact of moving away from Level 1 (no problems) in each HRQL dimension is characterized by the two-factor interaction term between the QLU-C10D levels and *TIME*. This enables a utility algorithm in which the effect of each level of each dimension is included as a decrement away from full health (which has a value of 1).

We analyzed the DCE data with STATA 13.0 [46] in two ways. The primary analysis used conditional logit models (Eq. 1), in which the utility of option *j* in choice set *s* for survey respondent *i* was assumed to be

1 $${U}_{isj}=\alpha {TIME}_{isj}+ \beta {X}_{isj}^{\mathrm{^{\prime}}}{TIME}_{isj}+{\varepsilon }_{isj}$$

*i* = 1, …, *I respondents; j* = situations A, B; *s* = 1, …, 960 choice sets.

Here, *α* is the utility associated with a life year,${X}_{isj}^{\prime}$ is a vector of dummy variables representing the levels of the QLU-C10D health state presented in option *j*, and *β* is the corresponding vector of utility weights associated with each level in each dimension within ${X}_{isj}^{\prime}$, for each life year. The error term ${\varepsilon }_{ isj}$ was assumed to have a Gumbel distribution.

Because each respondent assessed up to 16 choice pairs, we allowed for intra-individual correlation, using a clustered sandwich estimator to adjust the standard errors. We estimated utility decrements for each movement away from Level 1 (no problems) in each QLU-C10D dimension by dividing each *β* term by *α* [44], and used the delta method [47] in STATA to estimate standard errors and confidence intervals for these ratios.

We estimated two versions of Eq. 1. Model 1 included every decrement from the best level (i.e., Level 1, no problems) in each dimension within ${X}_{isj}^{\prime}$; thus, ${X}_{isj}^{\prime}$ contained 30 terms (i.e., 10 dimensions x (4-1) levels within each). Model 2 imposed a restriction of monotonicity in the levels of the dimensions of the QLU-C10D health state classification system by combining non-monotonic levels and re-estimating the model. Model 2 therefore included a reduced number of estimates in *β* (the vector of preference weights).

We conducted unweighted and weighted analyses for all models. In weighted analyses, sampling weights controlled for non-representativeness in measured respondent characteristics using the iterative proportional fitting algorithm (i.e., raking) proposed by Deming and Stephan [48], and implemented in STATA using the *ipfweight* command. Variance inflation due to weighting was assessed by calculating the percentage increase in the standard errors of the unweighted versus weighted coefficients.

We compared utilities derived from the Japanese QLU-C10D algorithm with those from other countries in two ways. We randomly generated 500 QLU-C10D health states, and scored each according to five country-specific algorithms, then plotted them by country, ordered them according to the Japanese values.

The following three data quality assessment metrics were assessed. We tallied the number of respondents who chose either all As or all Bs across the choice sets, then re-estimated weighted Model 2 with their data excluded. We considered the time respondents took to complete the survey. We divided respondents into deciles based on total survey time, ran a conditional logit on the DCE data in each decile, then graphed the pseudo-R^2^ and the number of statistically significant coefficients for each decile, interpreting low values on either indicator as suggesting relatively low quality data.

## Results

### Sample characteristics

As Fig. 1 shows, 3513 respondents entered the survey, 2662 (76%) of whom were within sampling quotas, consented and completed at least one choice pair, and 2435 (69%) completed all choice pairs. The data from these 2435 participants were included in analyses to assess representativeness and estimate the Japanese value set.

The sample characteristics (*n* = 2435) are compared to published Japanese general population characteristics in Table 1. Study participants were representative in terms of sex, age, and paid employment. Our study team discussed the type and degree of non-representativeness of the remaining variables and agreed to include four variables in raking (weighting): household income, education, health status (EQ-5D), and mental health (Kessler 6). The three remaining demographics that were non-representative were not included in raking for the following reasons: Region—the Japanese population is generally homogeneous across regions; Work status—correlated with household income, which was included in raking; Relationship status—differences per category were small (< 3.2%).

### Respondents’ perception of the DCE valuation task

Online Resource 3 details respondent perceptions of the DCE valuation task. In summary, 44% rated the health state presentation as ‘unclear’ or ‘very unclear,’ and 23% found it ‘clear’ or ‘very clear.’ Regarding the choice task, 66% found it ‘difficult’ or ‘very difficult’ to choose between pairs of health states, and only 7% found it ‘easy’ or ‘very easy.’ With regard to the strategy participants used to choose between pairs of health states, 32% focused on aspects highlighted in yellow, 26% considered most aspects, and 25% focused on just a few aspects. Of 103 participants provided additional detail on their strategy, length of survival time was considered by 65/103 (51%) when choosing between health states. Acceptability of burden to themselves was cited by 22/103 (17%), and burden to others was cited by 18/103 (14%). Specific symptoms (pain, appetite, sleep) were cited by a small number of respondents (8, 4, and 4, respectively).

### Data quality

Online Resource 4 details the data quality findings. When data from the 73 respondents who gave either all As or all Bs across their completed choice sets was excluded, there was little difference (max absolute difference of 0.0042) and no evidence of bias (mean difference of − 0.00054) in coefficient estimates. Median survey completion was 12.5 min, minimum 3.75 min, and maximum 69.33 min. Respondents in all completion time deciles sped up as they became more familiar with the choice task (Figure A). The fastest completion time decile yielded the least statistically significant coefficients (6/31) and the slowest two deciles yielded the most (26/31 and 25/31, respectively) (Figure B). While this suggested slower respondents produced less random data, the pseudo-R^2^ values were similar across deciles.

### DCE Results

**Fig. 2:**
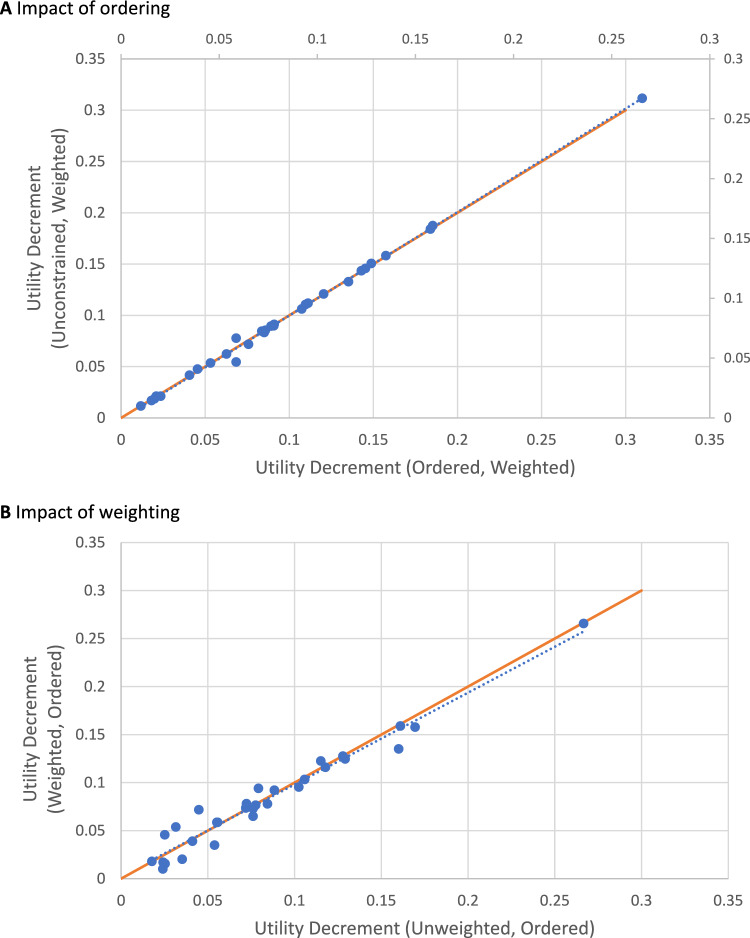
Impact of imposing monotonicity (ordering) and weighting: scatterplots of preference weights from conditional logit models (*n* = 2435)

Conditional logit results for the 2435 respondents who completed all 16 choice pairs are presented in Table 2. In the unweighted Model 1 analysis, all coefficients are negative and increase in absolute terms in progressively higher levels. Dimensions with the largest impact (based on the largest absolute coefficient) are physical functioning, pain, and role functioning. When responses were weighted, some small non-monotonicities were observed in the trouble sleeping dimension. The effect of combining levels to prevent this (Model 2) was small. Figure 2 shows the impact of enforcing monotonic ordering on the coefficients (Panel A) and using weights (Panel B). Both figures report a line of best fit between models with and without these adjustments, as well as a 45 degree line reporting equality. All data points are close to the 45 degree line, illustrating minimal impact of these adjustments, and thus the preference weights are robust to them. The standard errors of Model 1 coefficients in weighted analyses were on average 48% larger (minimum 27%, median 46%, maximum 77%). The combined effect of weighting on coefficient estimates and variance inflation reduced the level of statistical significance of three coefficients from 5% to non-significant (Social L2, Emotional L2, Pain L2), one from 1% to non-significant (Emotional L3), and three from 0.1% to 5% (Role level 2, Trouble Sleeping level 3, Nausea level 2). In one case, it increased statistical significance (Bowel problems from not significant to 5% to 1%).

**Table 2:** Conditional logit results for Model 1 (unconstrained) and Model 2 (monotonicity imposed), unweighted and weighted analyses (estimated coefficients and robust standard errors (SE)), based on data from respondents who completed all 16 choice pairs in the discrete choice experiment (*n* = 2435)

Coefficient^a^ (SE)	Level	Unweighted analysis	Weighted analyses^b^		Variance inflation^c^
Dimension		Model 1	Model 1	Model 2	
Duration	Linear (years)	0.4986 (0.0195)***	0.4769 (0.0345)***	0.4793 (0.0342)***	77%
Duration x Physical functioning	Level 2	− 0.0528 (0.0069)***	− 0.0494 (0.0102)***	− 0.0495 (0.0102)***	48%
	Level 3	− 0.0798 (0.0074)***	− 0.0646 (0.0102)***	− 0.0647 (0.0102)***	38%
	Level 4	− 0.1330 (0.0071)***	− 0.1273 (0.0101)***	− 0.1273 (0.0101)***	42%
Duration x Role Functioning	Level 2	− 0.0205 (0.0055)***	− 0.0194 (0.0079)*	− 0.0187 (0.0080)*	44%
	Level 3	− 0.0638 (0.0060)***	− 0.0615 (0.0080)***	− 0.0611 (0.0081)***	33%
	Level 4	− 0.0803 (0.0055)***	− 0.0766 (0.0070)***	− 0.0761 (0.0071)***	27%
Duration x Social functioning	Level 2	− 0.0120 (0.0054)*	− 0.0047 (0.0079)	− 0.0048 (0.0079)	46%
	Level 3	− 0.0441 (0.0058)***	− 0.0434 (0.0088)***	− 0.0441 (0.0087)***	52%
	Level 4	− 0.0574 (0.0054)***	− 0.0586 (0.0086)***	− 0.0587 (0.0086)***	59%
Duration x Emotional functioning	Level 2	− 0.0121 (0.0053)*	− 0.0076 (0.0073)	− 0.0082 (0.0073)	38%
	Level 3	− 0.0176 (0.0058)**	− 0.0086 (0.0082)	− 0.0097 (0.0080)	41%
	Level 4	− 0.0361 (0.0051)***	− 0.0373 (0.0074)***	− 0.0374 (0.0074)***	45%
Duration x Pain	Level 2	− 0.0127 (0.0055)*	-0.0069 (0.0081)	− 0.0074 (0.0081)	47%
	Level 3	− 0.0588 (0.0058)***	− 0.0543 (0.0089)***	− 0.0556 (0.0087)***	53%
	Level 4	− 0.0845 (0.0053)***	− 0.0752 (0.0084)***	-0.0756 (0.0083)***	58%
Duration x Fatigue	Level 2	− 0.0157 (0.0052)**	− 0.0255 (0.0080)**	− 0.0258 (0.0080)**	54%
	Level 3	− 0.0380 (0.0055)***	− 0.0340 (0.0085)***	− 0.0350 (0.0084)***	55%
	Level 4	− 0.0421 (0.0050)***	− 0.0367 (0.0075)***	− 0.0373 (0.0075)***	50%
Duration x Trouble sleeping	Level 2	− 0.0276 (0.0050)***	− 0.0317 (0.0069)***	− 0.0281 (0.0067)***	38%
	Level 3	− 0.0278 (0.0056)***	− 0.0223 (0.0088)*	− 0.0281 (0.0067)***	57%
	Level 4	− 0.0380 (0.0050)***	− 0.0293 (0.0072)***	− 0.0311 (0.0070)***	44%
Duration x Appetite	Level 2	− 0.0089 (0.0050)	− 0.0086 (0.0068)	− 0.0086 (0.0068)	36%
	Level 3	− 0.0359 (0.0055)***	− 0.0349 (0.0072)***	− 0.0352 (0.0072)***	31%
	Level 4	− 0.0387 (0.0050)***	− 0.0365 (0.0073)***	− 0.0367 (0.0073)***	46%
Duration x Nausea	Level 2	− 0.0269 (0.0052)***	− 0.0170 (0.0073)*	− 0.0167 (0.0073)*	40%
	Level 3	− 0.0511 (0.0056)***	− 0.0457 (0.0080)***	− 0.0457 (0.0080)***	43%
	Level 4	− 0.0644 (0.0051)***	− 0.0595 (0.0079)***	− 0.0597 (0.0079)***	55%
Duration x Bowel problems	Level 2	− 0.0126 (0.0051)*	− 0.0218 (0.0083)**	− 0.0219 (0.0083)**	63%
	Level 3	− 0.0224 (0.0056)***	− 0.0345 (0.0092)***	− 0.0344 (0.0092)***	64%
	Level 4	− 0.0395 (0.0050)***	− 0.0452 (0.0079)***	− 0.0451 (0.0079)***	58%
Pseudo R^2^		0.1199	0.1178	0.1177	Av. 48%
Log Pseudo-likelihood		− 23765	− 27536	− 27538	Min. 27%
Akaike information criterion (AIC)		47592	55133	55136	Med. 46%
Bayesian information criterion (BIC)		47879	55420	55414	Max.77%

^a^The coefficient for each level of each QOL domain was estimated as the interaction of that level with duration. Levels combined to ensure monotonicity within each dimension are noted in italics. Statistical significance: ***0.1%; **1%; *5%

^b^Analyses were weighted for four variables simultaneously using raking: income, education, health status (EQ-5D), mental health (Kessler 6)

^c^Variance inflation expressed as percentage increase in Model 1 coefficient SE = (weighted SE − unweighted SE)/unweighted SE; average (Av.), minimum (Min.), median (Med.), maximum (Max.)

As a further robustness check, the same analyses were run including all DCE data (*n* = 2,662 respondents who completed at least one choice set) and the subset who completed all 16 choice pairs and all subsequent demographics (*n* = 2,312). As shown in Online Resources 5 and 6, results for these subsets were very similar to those in Table 2 (*n* = 2435), i.e., the same to two decimal places in all cases and to three decimal places in most cases.

**Fig. 3:**
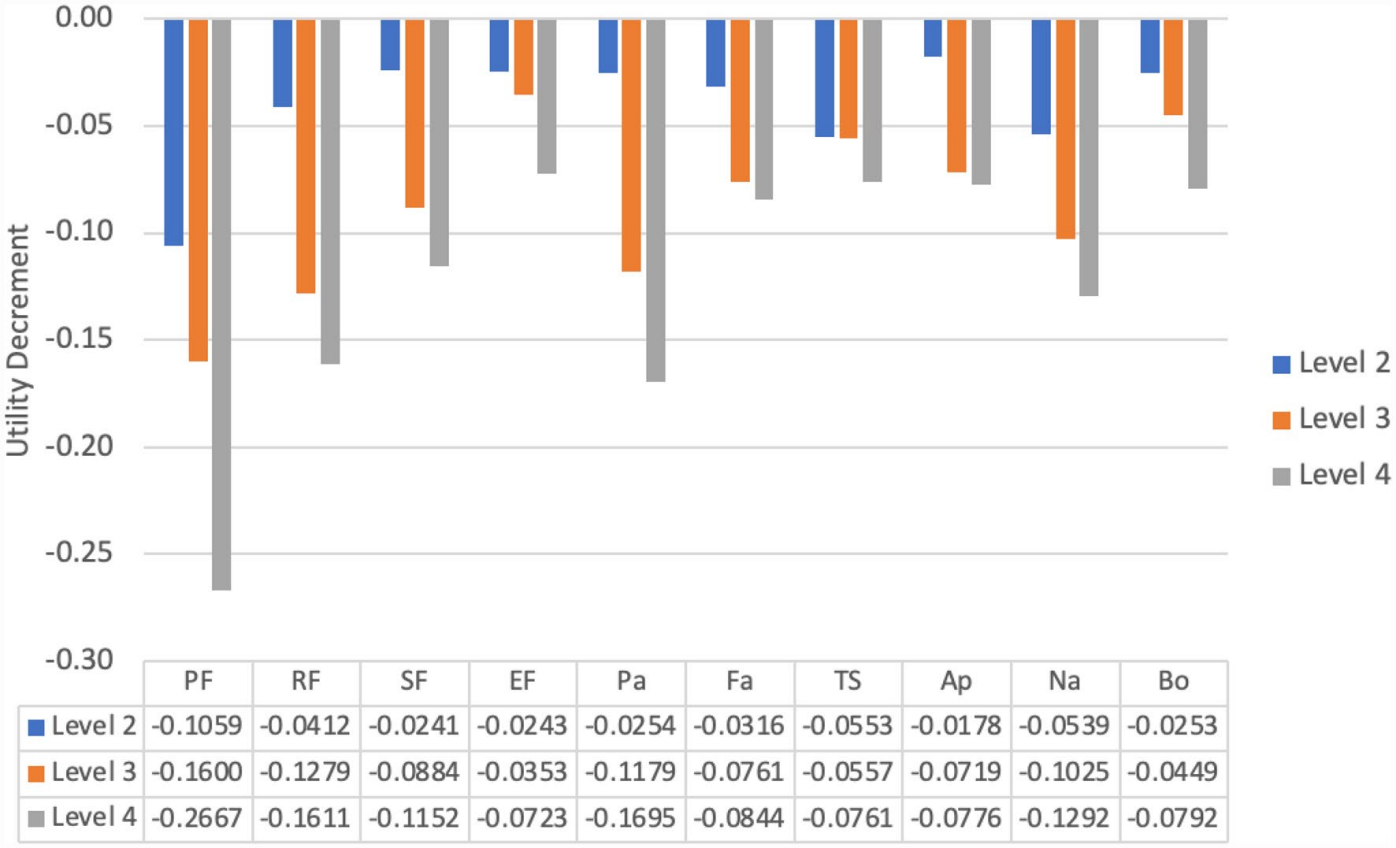
Japanese QLU-C10D preference weights for each dimension and level (Model 1 conditional logit, unweighted)

We calculated the QLU-C10D preference weights from the unweighted Model 1 results because they were fully monotonic, and because weighting did not change the coefficient estimates much but did increase standard errors considerably. These are plotted in Fig. 3 and tabulated under the graph. As these are derived by dividing through the coefficients in Table 2 by the duration coefficient, the pattern is unchanged, with physical functioning, pain, and role functioning the largest drivers of preference. We recommend these preference weights be used in the Japanese QLU-C10D scoring algorithm, provided in Online Resource 7, including syntax for STATA and SPSS.

### Japanese value set compared with other countries

**Fig. 4:**
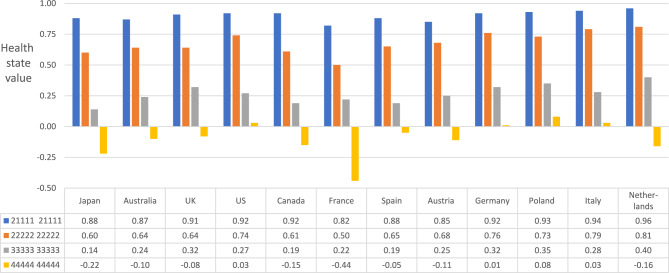
Comparison of Japanese utility scores for 4 health states with those using scoring algorithms from 11 other countries

The first comparison was based on four health states representing a range of health from very good to worst possible, with utility scores based on 12 country-specific utility algorithms (Fig. 4). For the best of these health states (with just a little physical functioning impairment and pain, 2111121111), the Japanese utility score ranked 8th of 12. For the health state with a little impairment in all domains (2222222222), the Japanese utility score ranked 11th, and for the health state with quite a bit of impairment in all domains (3333333333), the Japanese utility score was the lowest (rank 12/12). For the worst possible health state (very much impairment in all domains, 4444444444), the Japanese score (− 0.221) was ranked 11th, with only France having a lower value (− 0.44).

**Fig. 5:**
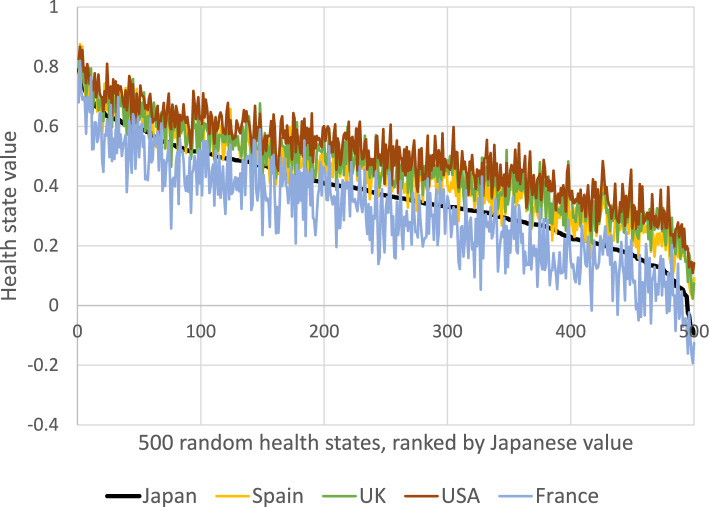
QLU-C10D health state values for Japan and five other countries

The second comparison, based on 500 randomly generated health states, compared the Japanese value set with two English-speaking countries (the United Kingdom (UK) and the United States (US) and two European countries (Spain and France) (Fig. 5). Across these health states, the Japanese values tend to lie above those from France, but below those from Spain, the UK, and the US. This suggests Japanese respondents were generally more willing to give up life expectancy for improved health than respondents in the latter three countries, but less likely than the French respondents. However, the pronounced oscillations in the lines for the four other countries indicate further complexity in the between-country story, due to variations between countries in dimension-specific preference weights.

## Discussion

This study provides the Japanese value set for the EORTC QLU-C10D, endorsed by the EORTC Quality of Life Group. The largest utility weights were associated with decrements in physical functioning, role functioning, and pain. Intermediate utility weights were associated with decrements in social functioning and nausea, while the remaining symptoms and emotional functioning were associated with smaller utility decrements. Compared with the QLU-C10D value sets from other countries, the Japanese decrements in social functioning, fatigue, and appetite were the largest of the 12 countries where QLU-C10D value sets have been established. The level 4 decrements in role function and nausea were the second largest among these countries. Generally, the Japanese weights of symptom-related items were larger than the average of the 12 countries, except for pain, where it was among the smaller. In addition, the comparison based on 500 randomly generated health states revealed considerable heterogeneity among countries, reflecting variations in dimension-specific preference weights in country-specific value sets. Different dimensions may play different roles in different cultures, but the observed variations may also be due in part to linguistic non-equivalence between countries; irrespective, these justify the need for country-specific value sets.

The value of the worst health state was -0.221, which was lower than that seen in most other existing QLU-C10D country-specific value sets, excluding France. This is a surprising result compared to the Japanese EQ-5D-5L value set. The worst Japanese EQ-5D-5L index [55555] was − 0.025, [49] which is the highest value in the world. This may be due to a key difference in the valuation methodologies used to generate the two value sets; the EORTC QLU-C10D was created by DCE with the duration method while the EQ-5D-5L was performed by composite time trade-off (cTTO) by the in-person interview. According to the Japanese EQ-5D-5L value set, the Japanese are reluctant to trade health states with death, suggesting a strong risk-aversion to death. By contrast, the international comparison of the EORTC QLU-C10D value set suggests that the Japanese willingly trade life-years with their health state. It may be caused by reflection of the Japanese preference; good health states are more preferable to long life years, but death is less acceptable, compared with Western people. Of course, it is possible that methodological artifacts may have contributed to the inconsistency. One of these is the method of preference elicitation: the QLU-C10D DCE was conducted as a self-complete survey while the EQ-5D-5L was interviewer administered. Another is translation effects in the source instrument (QLQ-C30 and EQ-5D-5L) and/or the preference elicitation DCE questionnaire for the QLU-C10D versus the cTTO for the EQ-5D-5L that somehow differentially distorted the Japanese preference task of the QLU-C10D relative to that of the EQ-5D-5L.

EQ-5D-5L is now a standard instrument for utility assessment in Japan. However, in clinical trial settings, the collection of EQ-5D-5L is sometimes omitted, and some studies include only disease-specific HRQL instruments. The QLQ-C30 and FACT-G are frequently used, particularly in cancer contexts [50]. Before the QLU-C10D was created, data obtained using the QLQ-C30 could not be used directly to calculate the QALY. Therefore, when QLQ-C30 data were used for cost-effectiveness analysis, mapping from the QLQ-C30 to PBMs was sometimes used. Although a mapping algorithm from the QLQ-C30 to the EQ-5D-5L has been established in Japan [51] mapping is not necessarily recommended for estimating utility, as there is considerable uncertainty around such calculations particularly at the extremes of the utility scale. In the Japanese HTA guidelines, mapping is only allowed if utility data cannot be obtained by other methods. As the QLU-C10D is now an established PBM, its use is more acceptable than that of the mapping algorithm. Also, because the EQ-5D-5L is a generic PBM, the QLQ-C30 is likely to be more sensitive to changes and differences in the health states of patients with cancer, and may therefore capture the utility of patients with cancer more appropriately and calculate the cost per QALY more precisely. Finally, many existing clinical trials have collected QLQ-C30 data in Japan. Such accumulated data can now be converted to utilities using the scoring algorithm generated by our research, and thereby provide HRQL weighting for QALY calculation. Given these advantages the Japanese value set for the EORTC QLU-C10D has many benefits for academics and the Japanese HTA system.

This study has several strengths. The Japanese value set was established in a large-sample representative of age and sex, enhancing generalisability of results. Second, only one inconsistency was observed in all the weights (the second and the third level of the “Trouble Sleeping” dimension). This is the lowest number of inconsistencies yet for QLU-C10D valuation studies, suggesting the Japanese survey was of high quality. Moreover, as our survey was based on the standard international protocol of the MAUCa Consortium, it facilitated international comparison with other country-specific QLU-C10D value sets. This study also has some limitations. Some respondents may not have engaged as fully in the online choice task as in face-to-face surveys. Also, respondents were not selected by random sampling from the entire Japanese population but by quota sampling from an online panel. Some characteristics of the respondents were statistically different from the Japanese population norms (e.g., region and work); we adjusted for most of these using raking, a form of sample weighting that allows weighting by several variables simultaneously. Further, the survey was conducted during the COVID-19 pandemic which had substantial impact on life in Japan. Other authors have noted that the pandemic did not impact on the ability to conduct online surveys such as this one during the pandemic, and indeed greater use and development of online research occurred during the pandemic [52]. However, it is unknown whether there was an impact on health preferences during the pandemic for the health attributes assessed in our study in comparison to pre- and post-pandemic preferences. There is limited evidence about the impacts of the COVID-19 pandemic on how people value health, and the policy implications of any such effects are unclear [53].

## Conclusion

This study employed data from approximately 2,500 Japanese general population respondents who completed a DCE task based on an international valuation protocol developed for the EORTC QLU-C10D by the MAUCa Consortium and the EORTC Quality of Life Group. This produced the EORTC-endorsed Japanese value set for the EORTC QLU-C10D, which has some distinguishing characteristics compared to existing country-specific QLU-C10D value sets. Fundamentally, this study promotes economic evaluations in Japan and the development of HTA systems that produce transparent, consistent and defensible decisions around health and healthcare.

### Supplementary Information

Below is the link to the electronic supplementary material.

Supplementary file1 (PDF 110 kb)

Supplementary file2 (PDF 120 kb)

Supplementary file3 (PDF 131 kb)

Supplementary file4 (PDF 117 kb)

Supplementary file5 (PDF 103 kb)

Supplementary file6 (PDF 126 kb)

Supplementary file7 (PDF 174 kb)

## Data Availability

The datasets generated and/or analyzed during the current study are not publicly available due to the lack of consent from participants, but are available from the corresponding author upon reasonable request.
