# Supplementary material for: Japanese value set for the EORTC QLU-C10D: A multi-attribute utility instrument based on the EORTC QLQ-C30 cancer-specific quality-of-life questionnaire
Source: Qual Life Res. 2024 May 9;33(7):1865–79. doi: 10.1007/s11136-024-03655-7 (PMC11176232; doi:10.1007/s11136-024-03655-7)
Supplement: Supplementary file 1 — Supplementary file1 (PDF 110 kb) [file 11136_2024_3655_MOESM1_ESM.pdf]

Japanese value set for the EORTC QLU-C10D: A multi-attribute utility instrument based on cancer-specific quality-of-life instrument.

Quality of Life Research

*Shiroiwa T[1], King MT[2,3], Norman R[4], Müller F[5,6], Campbell R[2], Kemmler G[3,7], Murata T[8], Shimozuma K[9], Fukuda T[1]

1. Center for Outcomes Research and Economic Evaluation for Health (C2H), National Institute of Public Health, Wako, Saitama, Japan
2. University of Sydney, Faculty of Science, School of Psychology, Sydney NSW, Australia
3. European Organisation for Research and Treatment of Cancer Quality of Life Group
4. School of Population Health, Curtin University, Perth, WA, Australia
5. Amsterdam UMC location University of Amsterdam, Medical Psychology, Meibergdreef 9, Amsterdam, Netherlands;
6. Amsterdam Public Health, Global Health, Amsterdam, Netherlands
7. Department of Psychiatry, Psychotherapy and Psychosomatics I, Medical University of Innsbruck, Innsbruck, Austria
8. Crecon Medical Assessment Co., Ltd., Tokyo, Japan
9. College of Life Sciences, Ritsumeikan University, Kusatsu, Japan

*Corresponding author:
Takeru Shiroiwa
Email: t.shiroiwa@icer.jp

Online resource 1 The QLU-C10D health state classification system, how it maps to the 13 component items from the QLQ-C30, and the duration attribute included the discrete choice experiment (DCE)

Dimension	Level	Stem	Descriptor	QLQ-C30 item scores
Physical Functioning[a,b]	1	You have…	No trouble taking a long walk outside of the house	Item 2 (long walk) = 1
	2		No trouble taking a short walk outside of the house, but at least a little trouble taking a long walk	Item 3 (short walk) = 1 AND Item 2 ≥ 2
	3		At least a little trouble taking a short walk outside of the house, and at least a little trouble taking a long walk	Item 3 = 2 AND Item 2 ≥ 2
	4		Quite a bit or very much trouble taking a short walk outside the house	Item 3 ≥ 3 AND Item 2 ≥ 2
Role Functioning	1	You are limited in pursuing your work or other daily activities…	Not at all	Item 6 = 1
	2		A little	Item 6 = 2
	3		Quite a bit	Item 6 = 3
	4		Very much	Item 6 = 4
Social Functioning[a,c]	1	Your physical condition or medical treatment interferes with your social or family life…	Not at all	Items 26 AND 27 = 1
	2		A little	Items 26 OR 27 = 2
	3		Quite a bit	Items 26 OR 27 = 3
	4		Very much	Items 26 OR 27 = 4
Emotional Functioning	1	You feel depressed…	Not at all	Item 24 = 1
	2		A little	Item 24 = 2
	3		Quite a bit	Item 24 = 3
	4		Very much	Item 24 = 4
Pain	1	You have pain…	Not at all	Item 9 = 1
	2		A little	Item 9 = 2
	3		Quite a bit	Item 9 = 3
	4		Very much	Item 9 = 4
Fatigue	1	You feel tired…	Not at all	Item 18 = 1
	2		A little	Item 18 = 2
	3		Quite a bit	Item 18 = 3
	4		Very much	Item 18 = 4
Sleep	1	You have trouble sleeping…	Not at all	Item 11 = 1
	2		A little	Item 11 = 2
	3		Quite a bit	Item 11 = 3
	4		Very much	Item 11 = 4
Appetite	1	You lack appetite…	Not at all	Item 13 = 1
	2		A little	Item 13 = 2
	3		Quite a bit	Item 13 = 3
	4		Very much	Item 13 = 4
Nausea	1	You feel nauseated…	Not at all	Item 14 = 1
	2		A little	Item 14 = 2
	3		Quite a bit	Item 14 = 3
	4		Very much	Item 14 = 4
Bowel Problems[a,c]	1	You…	do not have constipation or diarrhoea at all	Items 16 AND 17 = 1
	2		have a little constipation or diarrhoea	Items 16 OR 17 = 2
	3		have constipation or diarrhoea quite a bit	Items 16 OR 17 = 3
	4		have constipation or diarrhoea very much	Items 16 OR 17 = 4
Duration	1	You will live in this health state for…	1 year, and then die	Not applicable
	2		2 years, and then die	Not applicable
	3		5 years, and then die	Not applicable
	4		10 years, and then die	Not applicable

a. Three dimensions of the QLU-C10D each involve two QLQ-C30 items.
b. The Physical Functioning dimension includes ‘long walk’ and ‘short walk’ from the QLQ-C30; for the DCE, the levels were determined together, but were presented in DCE choice sets separately.
c. For Social Functioning and Bowel Problems, the QLU-C10D level is determined by the maximum value of the two component items.
