# Supplementary material for: Japanese value set for the EORTC QLU-C10D: A multi-attribute utility instrument based on the EORTC QLQ-C30 cancer-specific quality-of-life questionnaire
Source: Qual Life Res. 2024 May 9;33(7):1865–79. doi: 10.1007/s11136-024-03655-7 (PMC11176232; doi:10.1007/s11136-024-03655-7)
Supplement: Supplementary file 2 — Supplementary file2 (PDF 120 kb) [file 11136_2024_3655_MOESM2_ESM.pdf]

**Japanese value set for the EORTC QLU-C10D: A multi-attribute utility instrument based on cancer-specific quality-of-life instrument.**

Quality of Life Research

*Shiroiwa T[1], King MT[2,3], Norman R[4], Müller F[5,6], Campbell R[2], Kemmler G[3,7], Murata T[8], Shimozuma K[9], Fukuda T[1]

1. Center for Outcomes Research and Economic Evaluation for Health (C2H), National Institute of Public Health, Wako, Saitama, Japan
2. University of Sydney, Faculty of Science, School of Psychology, Sydney NSW, Australia
3. European Organisation for Research and Treatment of Cancer Quality of Life Group
4. School of Population Health, Curtin University, Perth, WA, Australia
5. Amsterdam UMC location University of Amsterdam, Medical Psychology, Meibergdreef 9, Amsterdam, Netherlands;
6. Amsterdam Public Health, Global Health, Amsterdam, Netherlands
7. Department of Psychiatry, Psychotherapy and Psychosomatics I, Medical University of Innsbruck, Innsbruck, Austria
8. Crecon Medical Assessment Co., Ltd., Tokyo, Japan
9. College of Life Sciences, Ritsumeikan University, Kusatsu, Japan

*Corresponding author:
Takeru Shiroiwa
Email: t.shiroiwa@icer.jp

**Online Resource 2** An example choice set from the discrete choice experiment valuation task as seen by Japanese survey participants.

| | 健康状態A | 健康状態B |
|---|---|---|
| 長い距離を歩く際に | 問題はない | 少なくとも少しは問題がある |
| 短い距離を歩く際に、 | 問題はない | 問題はない |
| 仕事や日常活動をすることに支障がある | かなりある | 少しある |
| あなたの身体の状態や治療は社会生活や家族生活をさまたげている | かなりある | 非常に多くある |
| 落ち込んだ気分である | かなりある | 少しある |
| 痛みがある | 少しある | 少しある |
| 疲労を感じる | まったくない | まったくない |
| 睡眠に支障がある | かなりある | かなりある |
| 食欲がない | かなりある | かなりある |
| 吐き気がある | 非常に多くある | 非常に多くある |
| 便秘または下痢がある | まったくない | まったくない |
| この健康状態で生きるのは | 2年間で、その後に死亡する | 5年間で、その後に死亡する |
| どちらの健康状態がよりよいと思いますか? | ○ 健康状態A | ○ 健康状態B |
