# Supplementary material for: Japanese value set for the EORTC QLU-C10D: A multi-attribute utility instrument based on the EORTC QLQ-C30 cancer-specific quality-of-life questionnaire
Source: Qual Life Res. 2024 May 9;33(7):1865–79. doi: 10.1007/s11136-024-03655-7 (PMC11176232; doi:10.1007/s11136-024-03655-7)
Supplement: Supplementary file 3 — Supplementary file3 (PDF 131 kb) [file 11136_2024_3655_MOESM3_ESM.pdf]

Japanese value set for the EORTC QLU-C10D: A multi-attribute utility instrument based on cancer-specific quality-of-life instrument.

Quality of Life Research

*Shiroiwa T[1], King MT[2,3], Norman R[4], Müller F[5,6], Campbell R[2], Kemmler G[3,7], Murata T[8], Shimozuma K[9], Fukuda T[1]

1. Center for Outcomes Research and Economic Evaluation for Health (C2H), National Institute of Public Health, Wako, Saitama, Japan
2. University of Sydney, Faculty of Science, School of Psychology, Sydney NSW, Australia
3. European Organisation for Research and Treatment of Cancer Quality of Life Group
4. School of Population Health, Curtin University, Perth, WA, Australia
5. Amsterdam UMC location University of Amsterdam, Medical Psychology, Meibergdreef 9, Amsterdam, Netherlands;
6. Amsterdam Public Health, Global Health, Amsterdam, Netherlands
7. Department of Psychiatry, Psychotherapy and Psychosomatics I, Medical University of Innsbruck, Innsbruck, Austria
8. Crecon Medical Assessment Co., Ltd., Tokyo, Japan
9. College of Life Sciences, Ritsumeikan University, Kusatsu, Japan

*Corresponding author:
Takeru Shiroiwa
Email: t.shiroiwa@icer.jp

Online resource 3 Respondent perceptions of DCE valuation task

Survey question: Did you find the questions on these 16 screens easier or harder than most surveys you do?

Response	Frequency	Percent
Easier	139	5.7%
About the same	585	24.1%
Harder	1543	63.7%
I couldn't say	157	6.5%
**Total**	**2424**	**100%**

Survey question: How clear was the presentation of the health states?

Response	Frequency	Percent
Very unclear	166	6.8%
Unclear	908	37.5%
Neither clear nor unclear	793	32.7%
Clear	511	21.1%
Very clear	46	1.9%
**Total**	**2424**	**100%**

Survey question: How difficult was it to choose between the pairs of health states on each screen?

Response	Frequency	Percent
Very difficult	331	13.7
Difficult	1324	54.6
Neither easy nor difficult	590	24.3
Easy	151	6.2
Very easy	28	1.2
**Total**	**2424**	**100%**

Survey question: Did you have a strategy for choosing between the pairs of health states on each screen?

Response	Frequency	Percent
I did not have a strategy	188	7.8%
I focused on just a few aspects of the health states	622	25.7%
I focused on the aspects that were highlighted in yellow	770	31.8%
I considered most of the aspects	635	26.2%
I considered all of the aspects	186	7.7%
Other	23	0.9%
**Total**	**2424**	100.

All participants, including those who selected the response 'Other' could enter free text to describe the strategy they used; 103 participants provided usable data, as summarized in the table below.

Length of survival time was considered by the majority of these participants (n=65/103, 51%) when choosing between the health states. Acceptability of burden to themselves was cited by 22/103 (17%), burden to others was cited by 18/103 (14%). Pain, appetite and sleep with cited by 8, 4 and 4 people, respectively.

Coding of free text describing choice strategy	Frequency	Percent
Length of survival	65	51%
Acceptability of burden to themselves	22	17%
Burden to others	18	14%
Pain	8	6%
Appetite	4	3%
Sleep	4	3%
Priority of each attribute for themselves	2	2%
Corresponding factor to their own experience	1	1%
Other than their own experience	1	1%
Walk ability	1	1%
**Total**	**103**	
