# Supplementary material for: Japanese value set for the EORTC QLU-C10D: A multi-attribute utility instrument based on the EORTC QLQ-C30 cancer-specific quality-of-life questionnaire
Source: Qual Life Res. 2024 May 9;33(7):1865–79. doi: 10.1007/s11136-024-03655-7 (PMC11176232; doi:10.1007/s11136-024-03655-7)

Japanese value set for the EORTC QLU-C10D: A multi-attribute utility instrument based on cancer-specific quality-of-life instrument.

Quality of Life Research

*Shiroiwa T[1], King MT[2,3], Norman R[4], Müller F[5,6], Campbell R[2], Kemmler G[3,7], Murata T[8], Shimozuma K[9], Fukuda T[1]

1. Center for Outcomes Research and Economic Evaluation for Health (C2H), National Institute of Public Health, Wako, Saitama, Japan
2. University of Sydney, Faculty of Science, School of Psychology, Sydney NSW, Australia
3. European Organisation for Research and Treatment of Cancer Quality of Life Group
4. School of Population Health, Curtin University, Perth, WA, Australia
5. Amsterdam UMC location University of Amsterdam, Medical Psychology, Meibergdreef 9, Amsterdam, Netherlands;
6. Amsterdam Public Health, Global Health, Amsterdam, Netherlands
7. Department of Psychiatry, Psychotherapy and Psychosomatics I, Medical University of Innsbruck, Innsbruck, Austria
8. Crecon Medical Assessment Co., Ltd., Tokyo, Japan
9. College of Life Sciences, Ritsumeikan University, Kusatsu, Japan

*Corresponding author:
Takeru Shiroiwa
Email: t.shiroiwa@icer.jp

Online Resource 4 Data Quality Results

In total, 73 of the 2,619 people who completed at least two choice sets answered the same for every DCE task (i.e. gave either all A's or all B's across their completed choice sets); when the data from these 73 respondents were excluded and weighted Model 2 was re-estimated, there was little difference (max absolute difference of 0.0042) and no evidence of bias (mean difference of -0.00054). Nine of the 30 estimated coefficients were the same to 3 decimal places (DP), a further 15 were the same to 2 DP(with rounding), and the remaining 6 differed by 0.01 when rounded to 2 DP but differed by a maximum of only 0.0035.

Statistics on time for survey completion were: median 12 minutes 34 seconds (12'34"), interquartile range 8'57" to 16'3", minimum 3'44", maximum 69'21".

Figure A shows that respondents in all completion time deciles sped up as they became more familiar with the choice task.

Figure B shows the fastest completion time decile yielded the least statistically significant coefficients (6/31) and the slowest two deciles yielded the most (26/31 and 25/31, respectively). While this suggested slower respondents produced less random data, the pseudo R^2 values were similar across deciles.

Figure A Distribution of time to completion by choice set

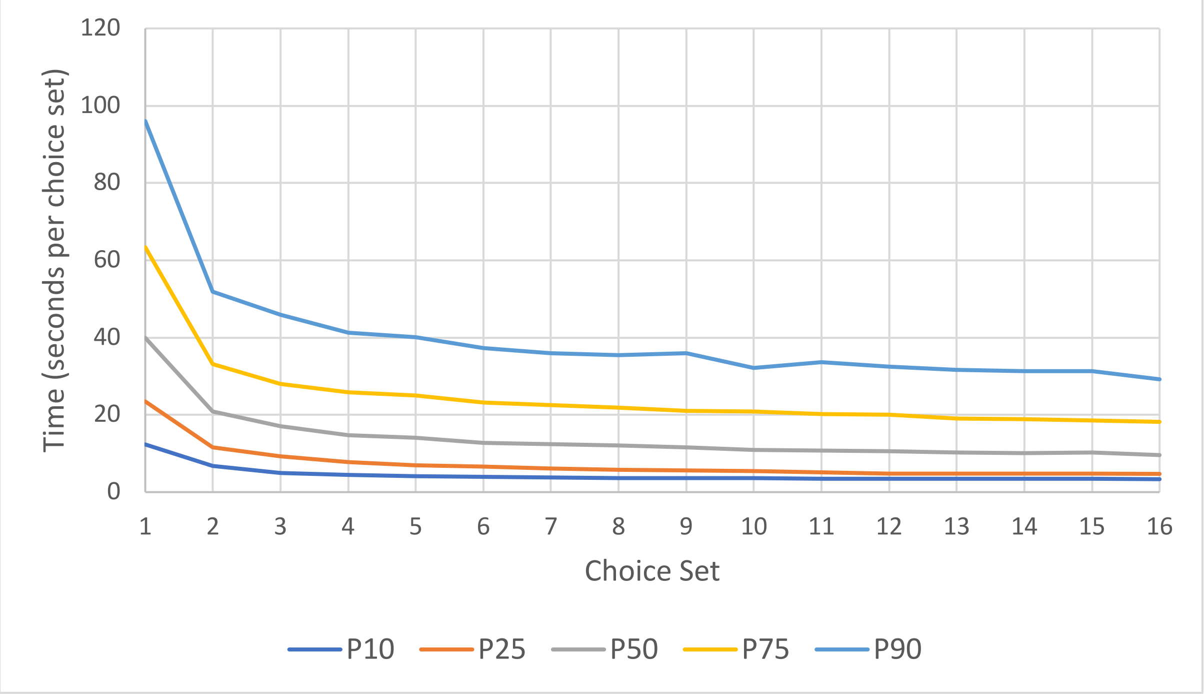

Figure B Relationship of completion time decile with model fit and number of statistically significant coefficients

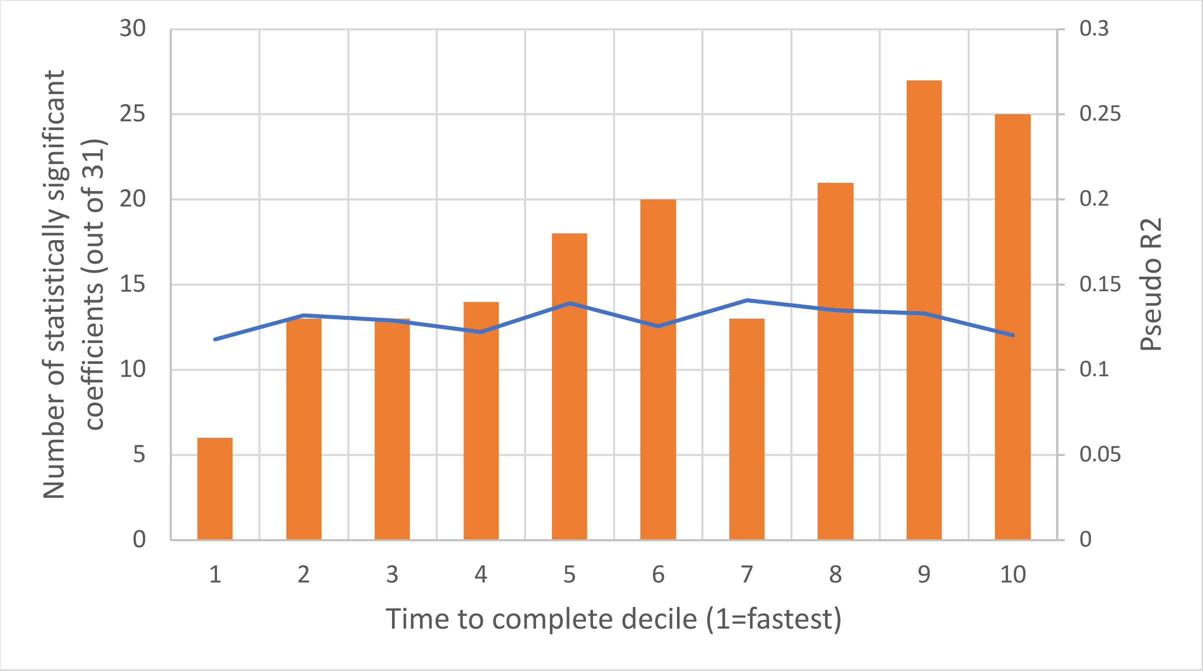

Supplement: Supplementary file 4 — Supplementary file4 (PDF 117 kb) [file 11136_2024_3655_MOESM4_ESM.pdf]
