# Supplementary material for: Japanese value set for the EORTC QLU-C10D: A multi-attribute utility instrument based on the EORTC QLQ-C30 cancer-specific quality-of-life questionnaire
Source: Qual Life Res. 2024 May 9;33(7):1865–79. doi: 10.1007/s11136-024-03655-7 (PMC11176232; doi:10.1007/s11136-024-03655-7)
Supplement: Supplementary file 5 — Supplementary file5 (PDF 103 kb) [file 11136_2024_3655_MOESM5_ESM.pdf]

Japanese value set for the EORTC QLU-C10D: A multi-attribute utility instrument based on cancer-specific quality-of-life instrument.

Quality of Life Research

*Shiroiwa T[1], King MT[2,3], Norman R[4], Müller F[5,6], Campbell R[2], Kemmler G[3,7], Murata T[8], Shimozuma K[9], Fukuda T[1]

1. Center for Outcomes Research and Economic Evaluation for Health (C2H), National Institute of Public Health, Wako, Saitama, Japan
2. University of Sydney, Faculty of Science, School of Psychology, Sydney NSW, Australia
3. European Organisation for Research and Treatment of Cancer Quality of Life Group
4. School of Population Health, Curtin University, Perth, WA, Australia
5. Amsterdam UMC location University of Amsterdam, Medical Psychology, Meibergdreef 9, Amsterdam, Netherlands;
6. Amsterdam Public Health, Global Health, Amsterdam, Netherlands
7. Department of Psychiatry, Psychotherapy and Psychosomatics I, Medical University of Innsbruck, Innsbruck, Austria
8. Crecon Medical Assessment Co., Ltd., Tokyo, Japan
9. College of Life Sciences, Ritsumeikan University, Kusatsu, Japan

*Corresponding author:
Takeru Shiroiwa
Email: t.shiroiwa@icer.jp

Online Resource 5 Conditional logit results for Model 1 (unconstrained) and Model 2 (monotonicity imposed), unweighted and weighted analyses (estimated coefficients and robust standard errors (SE)), based on data from respondents who completed the entire survey (i.e. all 16 choice sets and all demographic questions, n=2312)

Coefficient[a] (SE)		Unweighted Analysis	Weighted Analyses[b]	
Dimension	Level	Unconstrained	Unconstrained	Ordered
Duration	Linear (years)	0.4948 (0.0202)***	0.4703 (0.0359)***	0.4732 (0.0355)***
Duration x Physical Functioning	Level 2	-0.0519 (0.0071)***	-0.0461 (0.0105)***	-0.0463 (0.0105)***
	Level 3	-0.0781 (0.0077)***	-0.0620 (0.0105)***	-0.0621 (0.0105)***
	Level 4	-0.1321 (0.0073)***	-0.1250 (0.0104)***	-0.1249 (0.0104)***
Duration x Role Functioning	Level 2	-0.0211 (0.0057)***	-0.0207 (0.0082)*	-0.0199 (0.0082)*
	Level 3	-0.0635 (0.0061)***	-0.0614 (0.0083)***	-0.0609 (0.0084)***
	Level 4	-0.0801 (0.0057)***	-0.0755 (0.0073)***	-0.0749 (0.0073)***
Duration x Social Functioning	Level 2	-0.0120 (0.0056)*	-0.0041 (0.0082)	-0.0043 (0.0082)
	Level 3	-0.0437 (0.0061)***	-0.0416 (0.0091)***	-0.0425 (0.0090)***
	Level 4	-0.0582 (0.0056)***	-0.0581 (0.0089)***	-0.0582 (0.0089)***
Duration x Emotional Functioning	Level 2	-0.0116 (0.0055)*	-0.0057 (0.0075)	-0.0064 (0.0076)
	Level 3	-0.0156 (0.006)**	-0.0058 (0.0084)	-0.0071 (0.0083)
	Level 4	-0.0358 (0.0053)***	-0.0370 (0.0077)***	-0.0372 (0.0076)***
Duration x Pain	Level 2	-0.0120 (0.0057)*	-0.0079 (0.0084)	-0.0086 (0.0084)
	Level 3	-0.0586 (0.006)***	-0.0554 (0.0092)***	-0.0570 (0.0090)***
	Level 4	-0.0855 (0.0054)***	-0.0762 (0.0087)***	-0.0767 (0.0086)***
Duration x Fatigue	Level 2	-0.0154 (0.0053)**	-0.0248 (0.0084)**	-0.0252 (0.0084)**
	Level 3	-0.0378 (0.0056)***	-0.0333 (0.0089)***	-0.0345 (0.0087)***
	Level 4	-0.0419 (0.0052)***	-0.0364 (0.0078)***	-0.0371 (0.0077)***
Duration x Trouble Sleeping	Level 2	-0.0288 (0.0052)***	-0.0308 (0.0071)***	-0.0263 (0.0069)***
	Level 3	-0.0281 (0.0058)***	-0.0192 (0.0091)*	-0.0292 (0.0072)***
	Level 4	-0.0362 (0.0052)***	-0.0270 (0.0074)***	-0.0090 (0.0070)
Duration x Appetite	Level 2	-0.0088 (0.0052)	-0.0090 (0.0070)	-0.0329 (0.0074)***
	Level 3	-0.0330 (0.0056)***	-0.0325 (0.0075)***	-0.0381 (0.0076)***
	Level 4	-0.0394 (0.0052)***	-0.0379 (0.0076)***	-0.0162 (0.0076)*
Duration x Nausea	Level 2	-0.0264 (0.0054)***	-0.0166 (0.0075)*	-0.0453 (0.0083)***
	Level 3	-0.0506 (0.0058)***	-0.0453 (0.0083)***	-0.0583 (0.0083)***
	Level 4	-0.0621 (0.0052)***	-0.0581 (0.0083)***	-0.0218 (0.0086)*
Duration x Bowel Problems	Level 2	-0.0117 (0.0053)*	-0.0218 (0.0086)*	-0.0332 (0.0095)***
	Level 3	-0.0218 (0.0058)***	-0.0334 (0.0095)***	-0.0445 (0.0082)***
	Level 4	-0.0377 (0.0051)***	-0.0446 (0.0082)***	0.4732 (0.0355)***
Pseudo R^2		0.1198	0.1177	0.1176
Log Pseudo-likelihood		-22179	-26073	-26076
Akaike information criterion (AIC)		44420	52207	52212
Bayesian information criterion (BIC)		44705	52492	52488

a. The coefficient for each level of each QOL domain was estimated as the interaction of that level with duration. Levels combined to ensure monotonicity within each dimension are noted in italics

Levels of statistical significance: ***0.1%; **1%; *5%.

b. Analyses were weighted for four variables simultaneously using raking: income, education, health status (EQ-5D), mental health (Kessler 6).
