# Supplementary material for: Japanese value set for the EORTC QLU-C10D: A multi-attribute utility instrument based on the EORTC QLQ-C30 cancer-specific quality-of-life questionnaire
Source: Qual Life Res. 2024 May 9;33(7):1865–79. doi: 10.1007/s11136-024-03655-7 (PMC11176232; doi:10.1007/s11136-024-03655-7)
Supplement: Supplementary file 7 — Supplementary file7 (PDF 174 kb) [file 11136_2024_3655_MOESM7_ESM.pdf]

Japanese value set for the EORTC QLU-C10D: A multi-attribute utility instrument based on the EORTC QLQ-C30 cancer-specific quality-of-life questionnaire

Quality of Life Research

*Shiroiwa T[1], King MT[2,3], Norman R[4], Müller F[5,6], Campbell R[2], Kemmler G[3,7], Murata T[8], Shimozuma K[9], Fukuda T[1]

1. Center for Outcomes Research and Economic Evaluation for Health (C2H), National Institute of Public Health, Wako, Saitama, Japan
2. University of Sydney, Faculty of Science, School of Psychology, Sydney NSW, Australia
3. European Organisation for Research and Treatment of Cancer Quality of Life Group
4. School of Population Health, Curtin University, Perth, WA, Australia
5. Amsterdam UMC location University of Amsterdam, Medical Psychology, Meibergdreef 9, Amsterdam, Netherlands;
6. Amsterdam Public Health, Global Health, Amsterdam, Netherlands
7. Department of Psychiatry, Psychotherapy and Psychosomatics I, Medical University of Innsbruck, Innsbruck, Austria
8. Crecon Medical Assessment Co., Ltd., Tokyo, Japan
9. College of Life Sciences, Ritsumeikan University, Kusatsu, Japan

*Corresponding author:

Takeru Shiroiwa

Email: t.shiroiwa@icer.jp

Online resource 7

Instructions for calculating EORTC QLU-C10D scores from EORTC QLQ-C30 responses using Japanese general population preference weights, with STATA and SPSS code

To calculate the EORTC QLU-C10D score from EORTC QLQ-C30 responses of a particular patient p, first determine their QLU-C10D level l for each dimension d, following the mapping of QLQ-C30 items to QLU-C10D levels in Online Resource 1.

A utility score of 1 is assigned to patients whose QLQ-C30 scores indicate they are at level 1 of all 10 dimensions of the QLU-C10D. For all other health states, the utility score is 1 minus each the utility decrement (w_{dl}) for each level down from no problems in each of the 10 QLU-C10D dimensions.

STATA code to calculate EORTC QLU-C10D scores from EORTC QLQ-C30 responses, based on the Japanese utility weights

```
********************************************************************************
* Example code for converting EORTC QLQ-C30 data into QLU-C10D utility scores
* Written by Richard Norman, August 2023
*
* The utility algorithm reported in this code is based on the preference weights
* in Figure 3, which are derived from the ceofficients in the unweighted
* unconstrained conditional logit model in Table 2
*
* Any questions / comments on the instrument or code should be sent to
* Takeru Shiroiwa t.shiroiwa@icer.jp and richard.norman@curtin.edu.au
********************************************************************************
********************************************************************************
* Stage 1: Derive of the QLU-C10D dimension levels from the corresponding EORTC*
* QLQ-C30 item responses.
********************************************************************************
********************************************************************************
* Assumption: For this code to work, it is assumed that the EORTC QLQ-C30 code
* is set up as thirty columns, labelled qlq1-qlq30, each of which can take one
* of four values 1-4, where 1 = "Not at all", 2 = "A little", 3 = "Quite a
* bit", and 4 = "Very much". To derive the QLU-C10D, we only need 13 of these
* items, as described in as described in Online Resource 1.
*
* Seven of QLU-C10D items are single items from the EORTC QLQ-C30, and three
* (PF, SF, BO) are composite, combined as described below.
********************************************************************************
* Generate the QLU-C10D level for Physical Functioning from QLQ-C30 item 2
* (long walk) and item 3 (short walk)
gen pf=.
replace pf=1 if qlq2==1
replace pf=2 if qlq2>1 & qlq3==1
replace pf=3 if qlq2>1 & qlq3==2
replace pf=4 if qlq2>1 & qlq3==3
replace pf=4 if qlq2>1 & qlq3==4

*PF note: Level 1 is no problems in a long walk (and logically also in a short
* walk). Level 2 is any problem taking a long walk but no problem taking a short *
walk. Level 3 is a little problem with
a short walk (and a logically a little * or more problem with a long walk), and
level 4 is higher level problems (quite * a bit or very much) with both short and
long walk.
* Generate the QLU-C10D level for Role Functioning from QLQ-C30 item 6
* (work and daily activities)
gen rf=.
replace rf=1 if qlq6==1
replace rf=2 if qlq6==2
replace rf=3 if qlq6==3
replace rf=4 if qlq6==4
* Generate the QLU-C10D level for Social Functioning from QLQ-C30 item 26
```

```
* (family life) and item 27 (social activities)
gen sf=.
replace sf=1 if qlq26==1 & qlq27==1
replace sf=2 if qlq26==2 | qlq27==2
replace sf=3 if qlq26==3 | qlq27==3
replace sf=4 if qlq26==4 | qlq27==4
* SF note: SF in the QLU-C10D effectively uses the maximum value of either
* qlq26 or qlq27. So if qlq26 is 4 and qlq27 is 1, the utility dimension is at
* level 4. The vertical bar in the code signifies OR.
* Generate the QLU-C10D level for Emotional Functioning from QLQ-C30 item 24
* (depressed)
gen ef=.
replace ef=1 if qlq24==1
replace ef=2 if qlq24==2
replace ef=3 if qlq24==3
replace ef=4 if qlq24==4
* Generate the QLU-C10D level for Pain from QLQ-C30 item 9 (pain)
gen pa=.
replace pa=1 if qlq9==1
replace pa=2 if qlq9==2
replace pa=3 if qlq9==3
replace pa=4 if qlq9==4
* Generate the QLU-C10D level for Fatigue from QLQ-C30 item 18 (tired)
gen fa=.
replace fa=1 if qlq18==1
replace fa=2 if qlq18==2
replace fa=3 if qlq18==3
replace fa=4 if qlq18==4
* Generate the QLU-C10D level for Sleep from QLQ-C30 item 11 (trouble sleeping)
gen sl=.
replace sl=1 if qlq11==1
replace sl=2 if qlq11==2
replace sl=3 if qlq11==3
replace sl=4 if qlq11==4
* Generate the QLU-C10D level for Appetite from QLQ-C30 item 13 (lack appetite)
gen ap=.
replace ap=1 if qlq13==1
replace ap=2 if qlq13==2
replace ap=3 if qlq13==3
replace ap=4 if qlq13==4
* Generate the QLU-C10D level for Nausea from QLQ-C30 item 14 (nauseated)
gen na=.
replace na=1 if qlq14==1
replace na=2 if qlq14==2
replace na=3 if qlq14==3
replace na=4 if qlq14==4
* Generate the QLU-C10D level for Bowel Problems from QLQ-C30 item 16 (constipated)
* and item 17 (diarrhea)
gen bo=.
replace bo=1 if qlq16==1 & qlq17==1
replace bo=2 if qlq16==2 | qlq17==2
replace bo=3 if qlq16==3 | qlq17==3
replace bo=4 if qlq16==4 | qlq17==4
* BO note: BO in the QLU-C10D effectively uses the maximum value of either
* qlq16 or qlq17. So if qlq16 is 4 and qlq17 is 1, the utility dimension is at
* level 4.
*******************************************************************************
* Stage 2: Generate utility decrements and sum to estimate utility scores
* The utility decrements (e.g. pfdec is the utility decrement for Physical
* Functioning dimension) are derived from Figure 3 and are derived
* from a conditional logit that was unconstrained and unweighted.
*******************************************************************************
gen pfdec=0
replace pfdec=-0.1059 if pf==2
replace pfdec=-0.1600 if pf==3
replace pfdec=-0.2667 if pf==4
gen rfdec=0
replace rfdec=-0.0412 if rf==2
```

```
replace rfdec=-0.1279 if rf==3
replace rfdec=-0.1611 if rf==4
gen sfdec=0
replace sfdec=-0.0241 if sf==2
replace sfdec=-0.0884 if sf==3
replace sfdec=-0.1152 if sf==4
gen efdec=0
replace efdec=-0.0243 if ef==2
replace efdec=-0.0353 if ef==3
replace efdec=-0.0723 if ef==4
gen padec=0
replace padec=-0.0254 if pa==2
replace padec=-0.1179 if pa==3
replace padec=-0.1659 if pa==4
gen fadec=0
replace fadec=-0.0316 if fa==2
replace fadec=-0.0761 if fa==3
replace fadec=-0.0844 if fa==4
gen sldec=0
replace sldec=-0.0553 if sl==2
replace sldec=-0.0557 if sl==3
replace sldec=-0.0761 if sl==4
gen apdec=0
replace apdec=-0.0178 if ap==2
replace apdec=-0.0719 if ap==3
replace apdec=-0.0776 if ap==4
gen nadec=0
replace nadec=-0.0539 if na==2
replace nadec=-0.1025 if na==3
replace nadec=-0.1292 if na==4
gen bodec=0
replace bodec=-0.0253 if bo==2
replace bodec=-0.0449 if bo==3
replace bodec=-0.0792 if bo==4
* Generate the QLU-C10D utility score
gen qluc10d = 1+pfdec+rfdec+sfdec+efdec+padec+fadec+sldec+apdec+nadec+bodec
replace qluc10d=. if pf==.|sf==.|ef==.|pa==.|fa==.|sl==.|ap==.|na==.|bo==.
*******************************************************************************
* The new variable qluc10d is a utility score where full health (i.e. level 1
* in each of the utility levels) is scored at 1. These data can now be used to
* construct quality-adjusted life years (QALYs) for cost-utility analysis.
*******************************************************************************
```

SPSS code to calculate EORTC QLU-C10D scores from EORTC QLQ-C30 responses, based on the Japanese utility weights

```
*****************************************************************************
* Example code for converting EORTC QLQ-C30 data into QLU-C10D utility scores  *
* Written for SPSS by Daniel Costa, October 2017                               *
* Adapted in 2023 for the Japanese value set                             *
*
* The utility algorithm in this code is based the preference weights
* in Figure 3, which are derived from the coefficients in the unweighted
* unconstrained conditional logit model in Table 2.
*
* This code is written for SPSS users, and notes are added throughout to allow*
* conversion to other software as required.
*
* Any questions / comments on the instrument or code should be sent to         *
* Takeru Shiroiwa t.shiroiwa@icer.jp and richard.norman@curtin.edu.au
*
*****************************************************************************

*****************************************************************************
* Stage 1: Derive the QLU-C10D dimension levels from the corresponding EORTC   *
* QLQ-C30 item responses.                    *
*****************************************************************************

*****************************************************************************
* Assumption: For this code to work, it is assumed that the EORTC QLQ-C30 code *
* is set up as thirty columns, labelled qlq1-qlq30, each of which can take one *
* of four values 1-4, where 1 = "Not at all", 2 = "A little", 3 = ?Quite a     *
* bit?, and 4 = ?Very much". To derive the QLU-C10D, we only need 13 of these  *
* items, as described in Online Resource 1.
*                  *
* Seven of QLU_C10D items are single items from the EORTC QLQ-C30, and three   *
* (PF, SF, BO) are composite, combined as described below.                     *
*****************************************************************************

* Generate the QLU-C10D level for Physical Functioning from QLQ-C30 item 2
* (long walk) and item 3 (short walk).
compute pf=$sysmis.
if qlq2=1 pf=1.
if qlq2>1 pf=2.
if qlq3>1 pf=3.
if qlq3>2 pf=4.
exe.


*PF note: Level 1 is no problems in a long walk (and logically also in a short
* walk). Level 2 is any problem taking a long walk but no problem taking a short
* walk. Level 3 is a little problem with a short walk (and a logically a little
* or more problem with a long walk), and level 4 is higher level problems (quite
* a bit or very much) with both short and long walk.

* Generate the QLU-C10D level for Role Functioning from QLQ-C30 item 6
* (work and daily activities).
compute rf=$sysmis.
if qlq6=1 rf=1.
if qlq6=2 rf=2.
if qlq6=3 rf=3.
if qlq6=4 rf=4.
exe.


* Generate the QLU-C10D level for Social Functioning from QLQ-C30 item 26
* (family life) and item 27 (social activities).
compute sf=$sysmis.
if qlq26=1 & qlq27=1 sf=1.
if qlq26=2 | qlq27=2 sf=2.
```

```
if qlq26=3 | qlq27=3 sf=3.
if qlq26=4 | qlq27=4 sf=4.
exe.

* SF note: SF in the QLU-C10D effectively uses the maximum value of either
* qlq26 or qlq27. So if qlq26 is 4 and qlq27 is 1, the utility dimension is at
* level 4.  The vertical bar in the code signifies OR.

* Generate the QLU-C10D level for Emotional Functioning from QLQ-C30 item 24
* (depressed).
compute ef=$sysmis.
if qlq24=1 ef=1.
if qlq24=2 ef=2.
if qlq24=3 ef=3.
if qlq24=4 ef=4.
exe.

* Generate the QLU-C10D level for Pain from QLQ-C30 item 9 (pain).
compute pa=$sysmis.
if qlq9=1 pa=1.
if qlq9=2 pa=2.
if qlq9=3 pa=3.
if qlq9=4 pa=4.
exe.

* Generate the QLU-C10D level for Fatigue from QLQ-C30 item 18 (tired).
compute fa=$sysmis.
if qlq18=1 fa=1.
if qlq18=2 fa=2.
if qlq18=3 fa=3.
if qlq18=4 fa=4.
exe.

* Generate the QLU-C10D level for Sleep from QLQ-C30 item 11 (trouble sleeping).
compute sl=$sysmis.
if qlq11=1 sl=1.
if qlq11=2 sl=2.
if qlq11=3 sl=3.
if qlq11=4 sl=4.
exe.

* Generate the QLU-C10D level for Appetite from QLQ-C30 item 13 (lack appetite).
compute ap=$sysmis.
if qlq13=1 ap=1.
if qlq13=2 ap=2.
if qlq13=3 ap=3.
if qlq13=4 ap=4.
exe.

* Generate the QLU-C10D level for Nausea from QLQ-C30 item 14 (nauseated).
compute na=$sysmis.
if qlq14=1 na=1.
if qlq14=2 na=2.
if qlq14=3 na=3.
if qlq14=4 na=4.
exe.

* Generate the QLU-C10D level for Bowel Problems from QLQ-C30 item 16 (constipated)
and item 17 (diarrhea).
compute bo=$sysmis.
if qlq16=1 & qlq17=1 bo=1.
if qlq16=2 | qlq17=2 bo=2.
if qlq16=3 | qlq17=3 bo=3.
if qlq16=4 | qlq17=4 bo=4.
exe.

* BO note: BO in the QLU-C10D effectively uses the maximum value of either
* qlq16 or qlq17. So if qlq16 is 4 and qlq17 is 1, the utility dimension is at
```

```
* level 4. The vertical bar in the code signifies OR.


********************************************************************************
* Stage 2: Generate utility decrements and sum to estimate utility scores
* The utility decrements (e.g. pfdec is the utility decrement for Physical
* Functioning dimension) are derived from Figure 3 and are derived
* from a conditional logit that was unconstrained and unweighted.
********************************************************************************
compute pfdec=0.
if pf=2 pfdec=-0.1059.
if pf=3 pfdec=-0.1600.
if pf=4 pfdec=-0.2667.
exe.

compute rfdec=0.
if rf=2 rfdec=-0.0412.
if rf=3 rfdec=-0.1279.
if rf=4 rfdec=-0.1611.
exe.

compute sfdec=0.
if sf=2 sfdec=-0.0241.
if sf=3 sfdec=-0.0884.
if sf=4 sfdec=-0.1152.
exe.

compute efdec=0.
if ef=2 efdec=-0.0243.
if ef=3 efdec=-0.0353.
if ef=4 efdec=-0.0723.
exe.

compute padec=0.
if pa=2 padec=-0.0254.
if pa=3 padec=-0.1179.
if pa=4 padec=-0.1695.
exe.

compute fadec=0.
if fa=2 fadec=-0.0316.
if fa=3 fadec=-0.0761.
if fa=4 fadec=-0.0844.
exe.

compute sldec=0.
if sl=2 sldec=-0.0553.
if sl=3 sldec=-0.0577.
if sl=4 sldec=-0.0761.
exe.

compute apdec=0.
if ap=2 apdec=-0.0178.
if ap=3 apdec=-0.0719.
if ap=4 apdec=-0.0776.
exe.

compute nadec=0.
if na=2 nadec=-0.0539.
if na=3 nadec=-0.1025.
if na=4 nadec=-0.1292.
exe.

compute bodec=0.
if bo=2 bodec=-0.0253.
if bo=3 bodec=-0.0449.
if bo=4 bodec=-0.0792.
exe.
```

```
* Generate the QLU-C10D utility score
compute qluc10d = 1+pfdec+rfdec+sfdec+efdec+padec+fadec+sldec+apdec+nadec+bodec.

if (sysmis(pf) or sysmis(sf) or sysmis(ef) or sysmis(pa) or sysmis(fa) or
sysmis(sl) or sysmis(ap) or sysmis(na) or sysmis(bo)) qluc10d=$sysmis.
exe.

* Show all decrement values to 3 decimal places.
formats pfdec rfdec sfdec efdec padec fadec sldec apdec nadec bodec qluc10d (F6.3).

******************************************************************************
* The new variable qluc10d is a utility score where full health (i.e. level 1 *
* in each of the utility levels) is scored at 1, and the minimum score (i.e.  *
* each utility level is at 4) is -0.095. These data can now be used to        *
* construct quality-adjusted life years (QALYs) for cost-utility analysis.
******************************************************************************
```
